# RET signaling in breast cancer therapeutic resistance and metastasis

**DOI:** 10.1186/s13058-023-01622-7

**Published:** 2023-03-14

**Authors:** Geoffrey Pecar, Simeng Liu, Jagmohan Hooda, Jennifer M. Atkinson, Steffi Oesterreich, Adrian V. Lee

**Affiliations:** 1 Women’s Cancer Research Center, UPMC Hillman Cancer Center and Magee-Womens Research Institute, Pittsburgh, PA USA; 2 Department of Pharmacology and Chemical Biology, University of Pittsburgh, The Assembly, Room 2051, 5051 Centre Avenue, Pittsburgh, PA 15213 USA; 3 School of Medicine, Tsinghua University, Beijing, China; 4 School of Medicine, University of Pittsburgh, Pittsburgh, PA USA

**Keywords:** RET, GDNF, Breast cancer, Metastasis, Drug resistance

## Abstract

RET, a single-pass receptor tyrosine kinase encoded on human chromosome 10, is well known to the field of developmental biology for its role in the ontogenesis of the central and enteric nervous systems and the kidney. In adults, RET alterations have been characterized as drivers of non-small cell lung cancer and multiple neuroendocrine neoplasms. In breast cancer, RET signaling networks have been shown to influence diverse functions including tumor development, metastasis, and therapeutic resistance. While RET is known to drive the development and progression of multiple solid tumors, therapeutic agents selectively targeting RET are relatively new, though multiple multi-kinase inhibitors have shown promise as RET inhibitors in the past; further, RET has been historically neglected as a potential therapeutic co-target in endocrine-refractory breast cancers despite mounting evidence for a key pathologic role and repeated description of a bi-directional relationship with the estrogen receptor, the principal driver of most breast tumors. Additionally, the recent discovery of RET enrichment in breast cancer brain metastases suggests a role for RET inhibition specific to advanced disease. This review assesses the status of research on RET in breast cancer and evaluates the therapeutic potential of RET-selective kinase inhibitors across major breast cancer subtypes.

**Supplementary Information:**

The online version contains supplementary material available at 10.1186/s13058-023-01622-7.

## Background

RET fills varied roles in vertebrate development, but serves few purposes in adult humans, with expression and function limited almost exclusively to protection of neural tissues in response to damage or inflammation [1]. RET alterations resulting in receptor hyperactivation have been identified as causal factors in multiple neoplastic diseases, most notably non-small cell lung carcinoma (NSCLC) [2] and both medullary and papillary thyroid carcinomas (MTC & PTC) [3, 4]. While RET expression in the normal human breast is negligible in both development and adulthood, RET has been shown to mediate multiple aspects of breast cancer development and progression, which are reviewed below [5–7].

In this review, the physiological function of RET in normal development is outlined and compared with current clinical and scientific appraisal of RET’s oncogenic contributions across multiple cancers and with a focus on breast cancer. Mechanistic details of RET-mediated signaling are provided, including key functions of the RET kinase domain and mechanisms of MAPK and PI3K/AKT/mTOR activation. The molecular role of RET as an oncogenic driver is then examined, including a summary discussion of RET alterations across cancers, with an emphasis on breast cancer-specific reports. RET is a key node in multiple breast cancer signaling networks. This review discusses in depth the interactions between RET and the key breast cancer drivers ERα and HER2, in addition to regulators of inflammatory response, cellular motility, and tumor growth. RET’s propensity to drive breast cancer metastasis, along with an emerging role for RET in brain-tropic metastatic colonization, highlight two critically important functions warranting further investigation. Finally, emerging therapeutic strategies for targeting RET are reviewed, with a particular interest in ongoing clinical trials emphasizing the expansion of access to highly selective RET inhibitors beyond NSCLC and thyroid cancers.

## Structure and function of RET, GDNF family ligands, and GFRα coreceptors

**Fig. 1:**
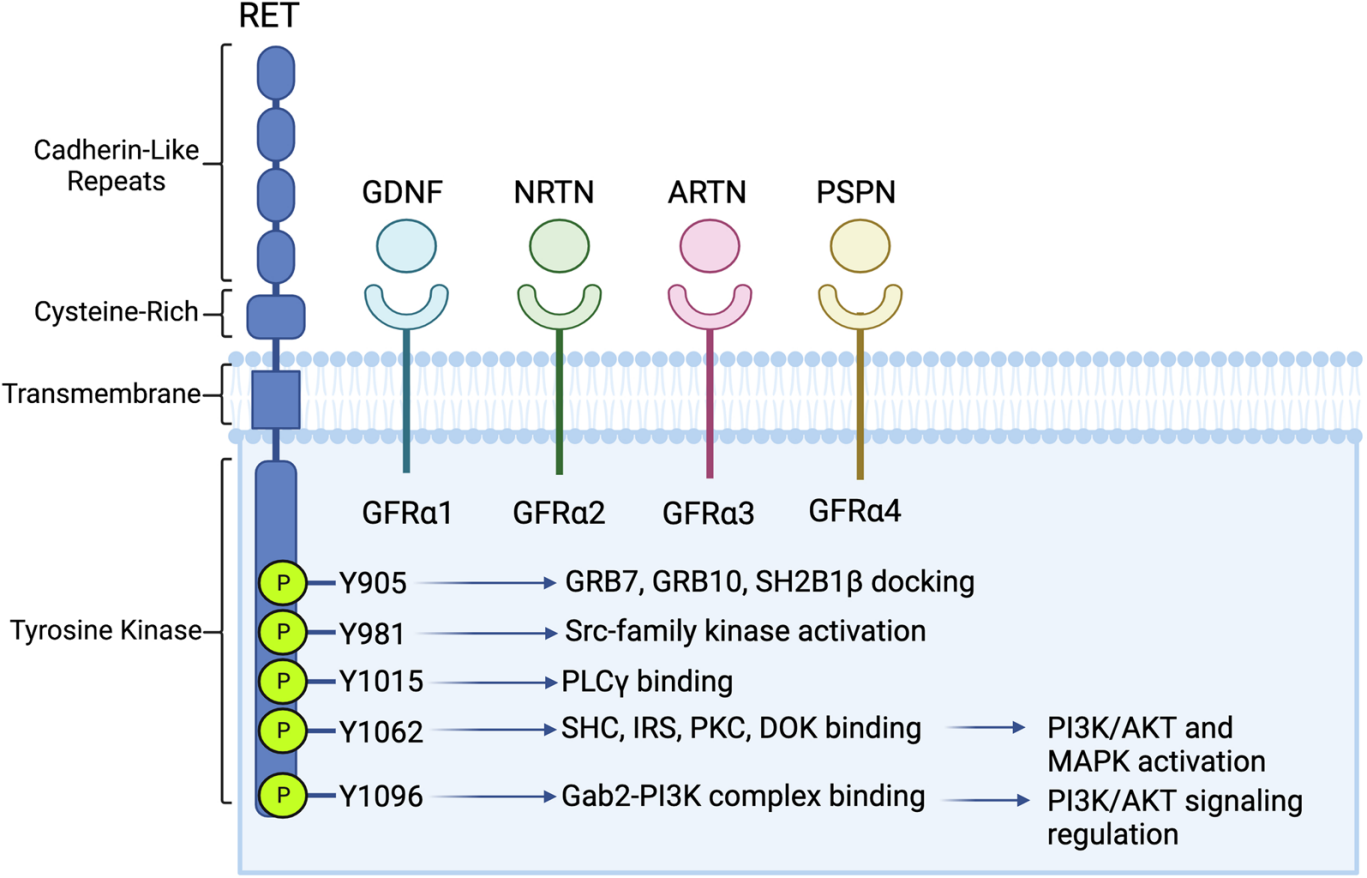
Schematic of RET structure, coreceptors, and corresponding ligands. Following GFL binding, RET-GFR-GFL complexes dimerize to activate intracellular signaling, facilitated by the adaptor and effector proteins, with critical functions indicated here and further reviewed in the above text

RET was originally discovered in 1985, following its identification as a transforming gene in NIH-3T3 cells transfected with lymphoma DNA [8]. From the time of its discovery, RET has been known as a unique oncogene with little homology to other known oncogenes, including *ERBB*, *SRC, FOS,* and *MYC* [8]. RET is distinguished from other members of the RTK superfamily by the presence of four extracellular cadherin domains, which lie adjacent to a cell surface-bound cysteine-rich domain [9] (Fig. 1). RET’s intracellular domains are multifunctional in the potentiation of signal transduction. The juxtamembrane segment contains phosphorylation sites which have been shown to rescue catalytically defective RET through allosteric inputs [10]. The kinase domain, comprising 339 residues beginning at L724, contains 11 tyrosine residues, of which 8 are capable of undergoing phosphorylation [11]. Alternative splicing results in the formation of three RET isoforms, which are marked by specific sequences of either 9 (RET9), 51 (RET51), or 43 (RET43) amino acids at the C-terminal tail. Both RET9 and RET51 can potentiate downstream kinase activation, however, RET51 is a more effective mediator of signal transduction due to distinct trafficking properties, including more efficient protein maturation and prolonged signaling activity before endosomal uptake [12]. Both the RET9 and RET51 isoforms have been shown to be expressed at varied levels in multiple breast cancer cell lines [13]; however, the specific protein trafficking and recycling observations described by Richardson et al. [12] have yet to be specifically examined in breast cancer models. While RET9 and RET51 have shown differing degrees of signal transduction capabilities in human cell lines, the RET43 splice form is a low-level transcript [14], and to our knowledge, no evidence exists for a function of RET43 in mammary tissue.

The glial cell-derived neurotrophic factor (GDNF) family receptors (GFRs) are key components of RET signaling, and act as direct receptors for RET ligands. These receptors, GFRα1, GFRα2, GFRα3, and GFRα4, bind the GDNF-family ligands (GFLs), with GFRα1, -2, -3, and -4 corresponding to GDNF, neurturin, artemin, and persephin, respectively. The GDNF-family ligands were initially purified in 1993 and have been shown to act as key growth factors in embryonic neurogenesis [15, 16]. The essential role of the GFRα family proteins in RET-mediated signal transduction has been recapitulated in breast cancer cell lines, with GFRα1 expression a limiting factor for GDNF-mediated signal activation in multiple in vitro studies [7, 13]. While the GFR coreceptors show a strong binding preference toward their corresponding ligands, the specificity of GFL-GFR interactions is not absolute [17]. The cascade of activity following GFL binding has been described using two competing models. In one model, GFL binding triggers the recruitment of a binary GFL-GFR complex to a RET monomer, followed by the formation of RET homodimers, transphosphorylation, and downstream kinase activation. An alternate proposal suggests that RET and GFR coreceptors are, to an extent, pre-associated on the cell membrane, and that ligand binding induces dimerization of extant RET-GFR complexes without the induction of a distinct coreceptor recruitment event [18].

### Mechanisms of RET-mediated signal transduction

RET-mediated signaling activates multiple intracellular pathways influencing processes which are critically dysfunctional in cancer, including mitosis, angiogenesis, and motility. Principal among the pathways mediated by RET are the mitogen-associated protein kinase (MAPK) and phosphatidylinositol-3-kinase (PI3K) pathways [19]. Following RET-GFR-GFL ternary complex formation and receptor dimerization, autophosphorylation of up to 14 of the 18 tyrosine residues of the intracellular domain may occur [20], and the specific functional implications of phosphorylation at several residues have been well-studied (Y905, Y981, Y1015, Y1062, and Y1096) (Fig. 1B) [9, 20].

Phosphorylation of each residue named above results in differential recruitment of intracellular adaptor and effector proteins related to the function of varied downstream pathways. The recruitment of intracellular substrates has been reviewed in detail by Arighi et al. [21] and most recently by Takahashi [20]. Briefly, Y905 serves as a docking site for GRB7, GRB10, and SH2B1*β* [20, 22, 23]; Y981 binds Src to mediate pro-survival signaling [24]; Y1015 mediates migratory signals via PLC-γ binding [25]; and Y1096 functions in the recruitment of Gab2-PI3K complexes and acts as a binding site for GRB2 [9, 26]. Y1062 acts as a polyfunctional residue, regulating the binding of multiple pathway effectors such as SHC family members, FRS2, IRS1/2, PKCα, DOK proteins, and Shank3 [27, 28]. The critical role of Y1062 in the downstream activation of the RAS-MAPK and PI3K-AKT pathways has been verified through single amino acid substitutions leading to loss of signal transduction [29]. While Y1062 is of central importance to RET-mediated MAPK signaling, Y1096 has been identified as an additional regulator of PI3K activation [30].

The MAPK, PI3K, and STAT pathways have been extensively characterized as drivers of cell growth and division across many cancer types, including breast cancer. While these pathways can be activated by a number of receptor tyrosine kinases, it is clear that RET can act as a key player in the mediation of these intracellular signaling pathways in breast-cancer specific settings [31, 32].

## The role of RET in ontogenesis

*RET* is most widely expressed in the embryonic stage and is best characterized for its influence on critical developmental processes such as neural crest cell migration and differentiation, kidney organogenesis, and development of the hindgut [15, 21, 33]. In the developing embryo, *RET* is first expressed around 9.5 days of gestation, with expression initially limited to the spinal cord, followed by expression in the spinal and cephalic ganglia, which persists until birth and ultimately drives the differentiation and axonal projection of dopaminergic neurons and the sympathetic nervous system. In these same neurons, glial cell line-derived neurotrophic factor (GDNF), a key component of RET signaling, has been shown to exert neuroprotective effects into adulthood [34, 35]. In the prenatal setting, RET’s functions begin to diversify as expression within the developing metanephros is exhibited during a brief window from 12.5 to 14.5 dpc [33]. RET protein is not expressed in the adult mammalian kidney despite an apparent key role in embryonic nephrogenesis.

With respect to developmental disorders, *RET* mutations are best characterized in the pathogenesis of Hirschsprung’s disease (HSCR), in which RET germline mutations and subsequent loss-of-function leads to aganglionosis of the colon with varying degrees of severity [36], accounting for roughly 50% of HSCR occurrences. Recently, somatic RET mutations have been identified as risk factors in HSCR, accounting for a further 18% of cases [37]. The central role of RET in the development of the excretory organs has been further demonstrated through the generation of *Ret* knockout mouse models, which exhibit defects such as kidney and ureter agenesis and reduced development of the enteric ganglia and reproductive system. Notably, mice homozygous for targeted *Ret* loss-of-function mutations die shortly after birth, further illustrating the critical role of RET in early survival [38, 39]. Critically, Vallone et al. have recently identified a role for RET in mammary gland development [40]. RET was shown to be highly expressed during lactation, with a reduction in expression observed during weaning-induced involution. Doxycycline-induced chronic overexpression of RET beyond the normal period of lactation was shown to induce multiple pathological characteristics, including hyperplasia of the mammary glands and intraepithelial metaplasia, suggesting a role for RET in early carcinogenesis.

## Oncogenic functions of RET

RET protein overexpression without gene amplification has been observed in 40–60% of breast tumors [41]. *RET* genomic alterations, however, are relatively rare in breast cancer, reported in approximately 1.2% of cases [42]. This lies in contrast to other RET-driven malignancies of the lung, thyroid, and colon, in which *RET* alterations are reported more frequently [3, 42–48]. Here, we review breast cancer-specific studies of *RET* alterations and where relevant, their relationships with specific breast cancer molecular subtypes and disease stages.

### Oncogenic RET overexpression in breast cancer

RET overexpression in the absence of gene amplification has been detected in 40–60% of breast tumors across multiple tumor subtypes [41]. In breast cancer, RET has been primarily studied in the context of estrogen-receptor positive (ER+) disease. ER is a central transcription factor in breast cancer, inducing both *RET* and *GFRA1* gene expression, which reciprocally function to enhance estrogen-driven cell proliferation [13]. While RET expression and function is significantly associated with ER positivity, RET overexpression has also been identified in the ER negative (ER−), triple negative (TN) and HER2-amplified breast cancer sub-groups [5, 49–51].

Gattelli and colleagues have shown that doxycycline-induced overexpression of the wild type RET51 isoform in mammary epithelium can generate ER-positive tumors in transgenic mouse models [5]. Administration of the multikinase inhibitor NVP-AST487 (an inhibitor of FLT3 which additionally inhibits RET, KDR, c-KIT and c-ABL at high concentrations) reduced tumor volume, corresponding to downregulation of MAPK, PI3K, and STAT1/3 [5]. While RET signaling through MAPK and PI3K have been well-characterized in various models, the consequences of RET-mediated STAT signaling warrant further investigation.

### RET fusions and point mutations in breast cancer

To date, there are relatively few studies of breast cancer-specific *RET* alterations. A large-scale study by Paratala et al. [42] revealed *RET* gene alterations in 121 of 9693 breast cancer samples (1.2%). Of the 121 reported alterations, 67% are *RET* gene amplifications, 20% are point mutations, and 13% are gene rearrangements [42], with an equal frequency of alterations between primary and metastatic tumor samples. *RET* rearrangements and gene amplifications were significantly associated with ER-negative samples, while *RET* missense point mutations were associated with ER-positivity. While there has been minimal research on RET’s function in ER-negative breast cancer to date, this finding suggests that activating *RET* mutations may warrant further study in this specific subtype of breast cancers.

The *RET* fusion genes described by Paratala et al. comprise *CCDC6-RET*, *NCOA4-RET* (which have previously been reported in NSCLC and PTC), and a previously undescribed *RASGEF1A-RET* fusion gene, all of which conferred constitutive kinase activity in multiple cell lines. Further, both *RET* amplification and *NCOA4-RET* fusions were shown to promote tumorigenesis in vivo. Finally, a patient harboring an *NCOA4-RET* fusion in an ER+/HER2+ bone metastatic lesion was treated with Cabozantinib as a means to target RET and showed a significantly positive therapeutic response. While the clinical portion of this study is inherently limited due to the inclusion of only one patient, these results highlight the potential efficacy of RET inhibition, and the need for increased surveillance of *RET* alterations in breast cancer primary tumors and recurrences. Given the frequency and clinical significance of bone metastases from breast cancer, the emergence of a *RET* fusion in a metastatic lesion of this type may also be a worthwhile area for future investigation.

An additional screening study of 4871 patient samples from cancers of diverse origin identified *RET* alterations in 3 of 506 breast cancer samples (a frequency of 0.6%). These alterations comprised 2 amplifications and 1 activating point mutation [52]. While the observations by Paratala et al. reviewed above make it clear that *RET* alterations may have important tumorigenic functions in breast cancer, this study places the full landscape of RET alterations in a broader context. For example, *RET* alterations were detected in 4 of 5 sampled medullary thyroid carcinomas (80%), 4 of 54 ovarian epithelial carcinomas (7.4%), and 27 of 527 lung carcinomas (5.1%), highlighting the rarity of RET alterations in breast cancer relative to other known RET-driven malignancies. Further, *RET* fusions have been identified with high frequency in other patient samples, including up to 17% of pancreatic carcinomas and 13% of cholangiocarcinomas [53]. While *RET* alterations occur at relatively high rates ni the cancers described above, evidence from studies of other cancer types (most notably salivary gland cancer [54, 55]) suggests that *RET* rearrangements and mutations, even when rare, may be associated with poor prognosis and should not be discounted as a critical topic of future research. Taken together, the studies above suggest that while infrequent in breast cancer, *RET* aberrations may present a promising therapeutic opportunity.

## RET signaling crosstalk with the estrogen receptor

### RET-ER crosstalk in breast cancer

The estrogen receptor (ER) is the central driver and key therapeutic target in approximately 70% of all breast cancers. RET expression is significantly correlated with ER-positivity in large-scale analyses of patient tissue, suggesting a specialized role for RET in ER+ breast cancer [7, 13, 56]. Multiple studies have examined the function of this relationship and have described the action of ER as a transcription factor regulating *RET* expression [57], signaling cross-talk between ER and RET [13, 58], and a role for RET in mediating endocrine therapy resistance [7, 59].

#### Estrogen regulation of RET expression

Multiple studies have noted elevated RET expression in ER-positive breast cancer cell lines, mirroring the observations from patient tissue described above [13, 60, 61]. In these cell lines, it has been consistently demonstrated that treatment with estradiol (E2) induces transcription of multiple RET signaling system components, including *RET*, *GFRA1*, and *ARTN*, suggesting a regulatory mechanism for RET’s functions in breast cancer. Interestingly, while GDNF expression is regulated by estrogens in the brain microenvironment to serve neuroprotective functions [62], to our knowledge this effect has not been recapitulated in breast cancer models.

While the evidence supporting estrogen regulation of RET and GFRA1 expression is abundant and clear, the relationship between RET expression and antiestrogen treatment remains to be completely deciphered. In multiple studies, treatment with Fulvestrant (ICI 182,780, or ICI) has been shown to reverse the E2-dependent induction of RET, GFRA1 and ARTN transcription. It has been demonstrated that Tamoxifen exerts a similar, but less complete, repression of RET expression, consistent with the effects of these drugs against other ER target genes [61, 63]. Research on the transcriptional effects of GDNF treatment in breast cancer models is extremely limited. However, Plaza-Menacho et al. demonstrated that in hormone deprived MCF-7 cells pre-treated with ICI, GDNF treatment significantly induced transcription of the ER target genes *FOS* and *CCND1* [58]. The reversal of this effect by combination ICI treatment suggests that GDNF provides a mechanism for the estrogen-independent activation of normally ER-mediated transcriptional programs. This effect was replicated in one study by Morandi et al. [7], in which estrogen-regulated genes were among those responsive to GDNF treatment in ICI-treated MCF7 cells, in addition to anti-apoptotic and pro-inflammatory pathways.

The regulatory network governing ER-induced *RET* expression in breast cancer models has been well-described with multiple estrogen response elements (EREs) identified within the *RET* enhancer region (where multiple databases note the presence of ER binding sites, reviewed in detail by Wang et al. [60])**.** This region, located at approximately − 50k Bp relative to the *RET* transcriptional start site, has been shown in two independent studies to act as a distal enhancer of RET expression in response to E2 treatment [57, 60]. Wang et al. provide a clear and detailed examination of the importance of FOXA1-ER crosstalk in the enhancement of RET transcription, illuminating a new role for a key transcription factor in breast cancer. In addition to further evidence for the role of FOXA1 in *RET* transcription, Stine et al. [57] demonstrate that retinoic acid (RA) is an additional regulator of *RET,* reproducing the influence of RA-mediated RET expression on embryonic development [64] and highlighting the synergy between ER and other transcriptional regulators in breast cancer.

#### The GDNF-RET axis regulates downstream ER signaling

The relationship between RET and ER extends beyond estrogen-regulated gene expression, and includes protein-level signaling interactions. Phosphorylation of ER is a key aspect of signal transduction in breast cancer, mediating functions such as transcription factor recruitment [65] and endocrine-refractory tumor growth [66]. Two studies by Plaza Menacho et al. [58] and Morandi et al. [7] have shown that GDNF stimulation of MCF7 breast cancer cells induces ER phosphorylation at residue S118, which has been associated with Tamoxifen resistance in vitro and relapse following Tamoxifen treatment in patient samples [67]. GDNF activates many intracellular pathways following binding to the RET-GFR signaling complex, and a few of these pathways have been demonstrated to mediate downstream ER phosphorylation. Comprehensive examination by Plaza-Menacho et al. [58] demonstrated that while P38, JNK, MEK, and PI3K directly contribute to this effect, mTOR serves as the chief intracellular signaling node governing the estrogen-independent, GDNF-induced phosphorylation of ER. These findings, in combination with the transcriptional effects described above, suggest that RET signaling might contribute to breast cancer endocrine resistance through multiple mechanisms.

#### RET as a driver of endocrine-resistant breast cancer

Gains in RET expression have been reported in multiple in vitro models of endocrine therapy-resistant breast cancer, including long-term estrogen deprived [7], tamoxifen-resistant [58, 59], and aromatase-overexpressing [68] breast cancer cell lines. In tamoxifen-resistant MCF-7 cell lines, increases in *GDNF* mRNA have been demonstrated in conjunction with depressed expression of hallmark ER target genes, suggesting that RET signaling is potentiated by ER-independent mechanisms in the presence of endocrine therapy [59].

Multiple studies have aimed to validate the function of RET signaling in endocrine resistance, and two mechanistic models have been characterized. Plaza-Menacho et al. in the earliest detailed examination of this RET-driven phenotype, showed that GDNF-RET signaling via PI3K and mTOR is a key molecular driver of endocrine resistance, and is significantly upregulated in MCF7-Tam-R cells relative to the parental cell line. siRNA-mediated RET knockdown not only eliminated GDNF-induced colony formation, but additionally sensitized MCF7-Tam-R cells to Tamoxifen, suggesting that RET expression is sufficient to induce endocrine resistance independent of GDNF treatment [58].

Subsequent studies have demonstrated an increase in RET expression in other models of antiestrogen resistance, but in contrast to the data reviewed above, have highlighted the importance of RET ligand expression and function rather than receptor overexpression alone. Morandi et al. [7] report that LTED conditioning of multiple breast cancer cell lines (MCF-7, T47D, and ZR75-1) results in increased RET expression and GDNF-mediated downstream signaling. Treatment with GDNF was sufficient to confer resistance to multiple aromatase inhibitors (letrozole, anastrozole, or exemestane) in parental cell lines, suggesting that abundance of the ligand alone, rather than RET receptor overexpression, is the limiting factor in RET-mediated endocrine resistance. Similarly, Horibata et al. [59] demonstrated that while RET expression is upregulated in multiple tamoxifen-resistant MCF-7 clones, treatment with GDNF induces resistance to both tamoxifen and fulvestrant in tamoxifen-sensitive MCF-7 cell lines. Artemin, the second-most abundant of the RET ligands, has also been shown to induce antiestrogen resistance. Artemin overexpression via stable transfection has been shown by Kang et al. to induce tumor growth and dissemination in vivo [69] and to induce resistance to both Tamoxifen in Fulvestrant in vitro [63], demonstrating that multiple components of the RET signaling system can modulate the endocrine-resistant breast cancer phenotype.

**Fig. 2:**
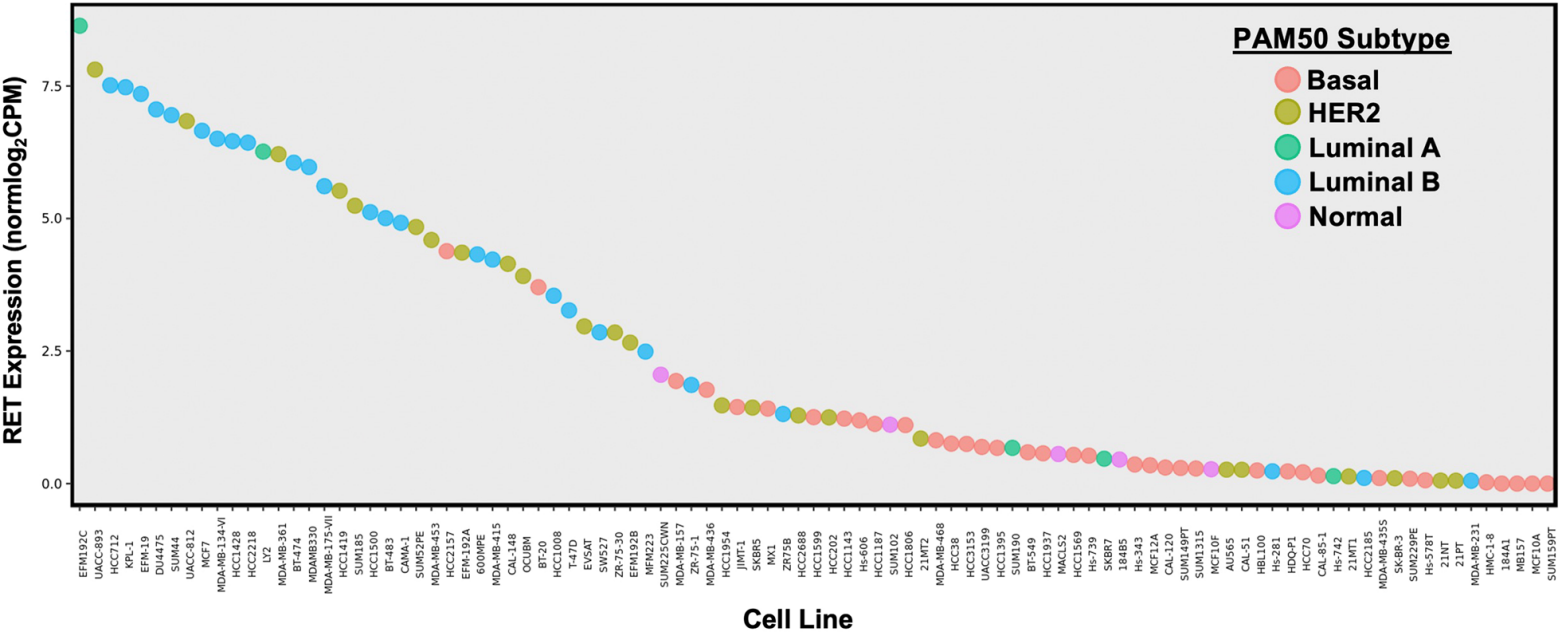
RET gene expression in breast cancer cell lines measured via RNASeq. Cell lines are ranked by RET Expression, and classified by cell line PAM50 subtype. Data were generated using RNA-seq fastqs using salmon (PMID: 28263959, v0.12.0, 31-kmer quasi-mapping, gcBias, seqBias, validateMappings). Fastqs from either (1) CCLE (PMID: 22460905), (2) Marcotte et al. Cell 2016 (PMID: 26771497), (3) courtesy of Dr. Joe Gray, OHSU. Mapping reference: Homo_sapiens.GRCh38.82. Expression represented as log2-transformed TMM-normalized CPMs. PAM50s called with genefu (PMID: 26607490). Full data are available to the public at LeeOesterreich.org

Validation of the in vitro and in vivo findings reviewed above using patient tumor samples and publicly available datasets suggests that both RET and GFL ligand expression are linked with endocrine resistance, but that ligand availability may be a crucial limiting factor. Morandi et al. [7] utilized estrogen-deprived MCF7 cells to generate a GDNF-response gene set (RGS) in the presence or absence of Fulvestrant pretreatment. Application of this analysis to a cohort of 52 ER+ breast cancer biopsy samples revealed a significant correlation between GDNF-RGS expression and letrozole resistance, with the highest GDNF-RGS occurring in Luminal B tumors, suggesting a relationship between RET activity and poor breast cancer prognosis. The clinical relevance of these findings was verified through the detection of increases in both RET expression and GDNF-regulated transcriptional pathways in recurrent tumor samples (73.1%) compared to 55.8% of primary tumors, which was reproduced in analysis of the TCGA and NIH ROCK databases. Taken together, these data suggest a role for RET expression and GDNF-mediated transcriptional programs in breast cancer disease progression, particularly in the aggressive Luminal B subtype. Notably, many Luminal B-like breast cancer cell lines are among those with the highest levels of RET mRNA expression, according to publicly available datasets (Fig. 2), though the function of RET outside ER+, HER2− breast cancer has yet to be examined in detail.

Horibata et al. [59] demonstrated that while RET expression correlates significantly with ER positivity, only 13% of the ER+ breast cancer samples in the TCGA dataset exhibit high RET ligand expression. A complementary examination of prospective biopsy microarrays revealed that while RET receptor expression is not significantly different between letrozole-responsive and resistant tumors, GFL ligand expression is significantly upregulated in cases of resistance.

A recent clinical study by Mechera et al. [56] in which 990 primary breast cancer tissues were screened for RET expression using immunohistochemistry has further confirmed the association between RET expression and ER positivity. While the highest frequency of RET expression (48.9%) was observed in HER2-positive Luminal-B primary tumors, differences in RET expression among ER+ subtypes was insignificant. Importantly, no significant relationship between primary tumor RET expression in univariate analysis of overall survival, suggesting that primary tumor RET expression is a limited in its utility as a prognostic marker in ER+ breast cancer.

While one cell line model has indicated a role for RET expression alone in the endocrine-resistant phenotype, the bulk of the findings above indicate that primary tumor expression of the GFL family ligands is the critical factor governing RET-mediated therapeutic resistance. Multiple studies have shown that the transcriptional and signaling relationships between ER and RET seem to result in their co-expression in patient tumors, yet it is clear that receptor expression alone is not currently a useful prognostic factor. However, the generation of GDNF-response signatures, in addition to the correlation between GFL ligand expression and endocrine therapy response, provide multiple avenues for future large-scale examination of patient tissues and generation of prognostic biomarkers. In sum, the data reviewed above suggest that RET signaling components may be promising biomarkers in endocrine-resistant tumors, and that RET inhibition may be a useful therapeutic strategy in a specific subset of patients. However, the relative importance of RET and its ligands in the generation of this disease phenotype is not yet completely understood.

While the reciprocal functional relationship between RET and ER has been relatively well-described, it is important to note that the bulk of the studies related to this phenomenon, and reviewed here, were published before the identification and characterization of multiple *ESR1* alterations (including hotspot mutations and gene fusions). While these are important areas of interest to breast cancer research and gains in RET expression have been reported in tumors or cell lines harboring ESR1 mutations [70, 71], and in cell line models of ESR1 fusions [72], relatively few studies have examined the specific influences of *ESR1* alterations on the RET-ER signaling and transcriptional axis.

## RET-HER2 signaling interactions present a challenge to breast cancer-targeted therapy

Using patient-derived xenograft models and cell lines, Gardaneh et al. [6] have demonstrated that GDNF-induced crosstalk between RET and HER2 is sufficient to confer resistance to HER2-targeting therapy. RET mRNA and protein expression was increased in PDX models derived from trastuzumab-resistant tumors, and treatment with GDNF was found to rescue Trastuzumab-sensitive cells both in vitro and in vivo following mammary fat pad injection. c-Src was identified as a key mediator of this signaling interaction, and inhibition of c-Src via treatment with saracatinib was sufficient to disrupt the GDNF-induced RET-HER2 signaling interaction in trastuzumab-resistant cell lines. While these findings have yet to be reproduced, the report of an association between RET expression and HER2-positive Luminal B tumors [56], in combination with this initial functional characterization, suggest that RET may be a useful target in multiple subtypes of breast cancer. Further, publicly available RNASeq data (Fig. 2) demonstrates the presence of RET expression in multiple HER2+ breast cancer cell lines, highlighting a potential area for future study.

## RET in breast cancer metastasis

### RET-FAK interactions promote breast cancer cell motility

Receptor tyrosine kinases (including HER2, IGF1R, and FGFR1) have previously been described as drivers of metastatic breast cancer phenotypes through aberrant activation of pro-migratory and pro-invasive signaling cascades in vitro. RET has previously been shown to activate cytoskeletal remodeling and migration by binding and phosphorylating FAK (PTK2) [73, 74] and has been reported to activate similar mechanisms in breast cancer models. Gattelli et al. [75] demonstrated that cell movement-related genes were among the most highly upregulated pathways following 6-day GDNF treatment in multiple breast cancer cell line models. RET-mediated FAK phosphorylation was identified as a key contributor to the migratory phenotype using the FAK inhibitor NVP-TAE836 and the multikinase inhibitor NVP-AST487. In vivo findings demonstrate that while RET inhibition did not affect the size of primary tumors, formation of lung metastases was reduced in mice treated with NVP-AST487. This corresponds to an accompanying analysis of an independent patient cohort, in which RET expression was negatively correlated with metastasis-free survival [75].

### RET as a potential driver of breast cancer brain metastasis

Breast cancer brain metastases (BCBMs) are rare at first diagnosis, but may affect up to 35% of patients with metastatic disease [76, 77], with a median patient survival as brief as 2.9 months under some therapeutic regimens [78]. BCBMs present an urgent clinical challenge, as therapeutic options have historically been limited to invasive surgery or radiotherapy due to the discordant drug sensitivity of central nervous system tumors. While a few HER2-targeting agents have shown a capability to control intracranial disease, the identification of new molecular targets for BCBM management remains a challenge to the field of breast cancer research.

A recent RNA sequencing study performed on patient-matched primary tumors and brain metastases suggests that RET may act as a driver of BCBM formation [79]. Using 20 paired samples processed for genome-wide exome capture RNASeq, RET was identified among the most consistently upregulated clinically actionable kinases. To validate a role for RET in BCBM, Varešlija et al. [80] examined the effects of RET inhibition in vitro and in vivo. In an ex vivo culture model of resected human BCBM, representing multiple breast cancer histologic subtypes, it was observed that treatment with either Cabozantinib (which inhibits RET along with MET, AXL, KIT, VEGFR and FLT3), or the pan-HER kinase inhibitor Afatinib was effective in the reduction of cell viability. Combination treatment did not produce an additive effect, suggesting RET inhibition alone may be sufficient for the treatment of RET-positive BCBM lesions. Subcutaneous implantation of a patient-derived BCBM xenograft into nude mice showed that either Afatinib or Cabozantinib was sufficient to neutralize tumor growth, further supporting RET inhibition as a possible strategy for BCBM therapy. While these preliminary results suggest RET may be an actionable therapeutic target in BCBM, the non-specific kinase inhibition provided by Cabozantinib is insufficient to isolate the role of RET signaling specifically.

RET signaling crosstalk has been reported in cell line models of other CNS tumors, including neuroblastoma [81]. RET is highly expressed in multiple neuroblastoma cell lines, along with TRK family receptors (TrkA and TrkB). While the neurotrophin ligands for the TRK family members (NGF, BDNF, and others) have not previously been shown to activate RET directly, Tetri et al. demonstrated that ARTN can trigger phosphorylation of TrkA, while NGF-mediated TrkA phosphorylation can trigger phosphorylation of RET [81]. Given this functional relationship with RET, past reports suggesting a functional role for TRK receptors in BCBM development [82], and the availability of potent dual RET/TRK inhibitors [83], crosstalk between RET and the TRK family receptors presents an opportunity for future investigation of signaling networks involved in BCBM.

## Inflammatory cytokines potentiate RET expression and signaling in breast cancer

The tumor microenvironment is a key regulator of cancer development and sustained tumor growth, with the expression of inflammatory cytokines acting as a contributor to carcinogenesis and therapeutic resistance in breast cancer [84, 85] as well as a negative prognostic factor [86]. In both cultured astrocytes and midbrain cell culture models, neurotrophic factors (including GDNF) have demonstrated regulation by inflammatory stimuli, illustrating the key role of RET signaling in neuroprotection [87, 88] Esseghir et al. [89] report that treatment with recombinant TNFα stimulates expression of *GDNF* mRNA in the NIH-3T3 cell line. Using MCF-7 xenografts, it was additionally shown that in vivo GDNF expression was specific to tumor-associated fibroblasts, and negligible in primary tumor cells. While this observation may suggest a relationship between GDNF expression and tumor environmental factors, detailed mechanistic studies of secreted factors are a technical challenge, and remain a limitation in this area of research.

**Fig. 3:**
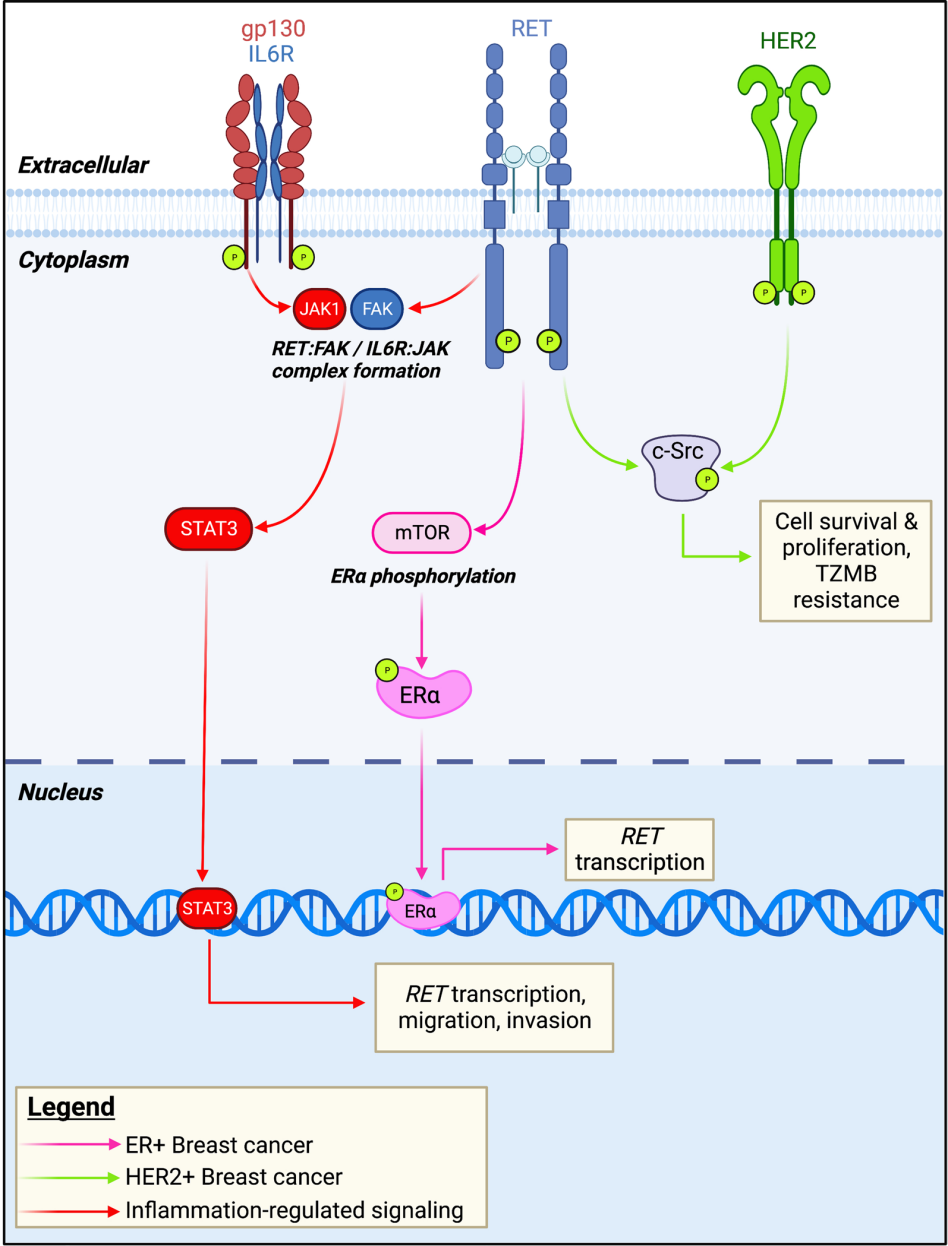
Key RET signaling and transcriptional interactions in breast cancer. Estrogen regulated gene expression is a key aspect of RET signaling function in ER-positive breast cancer. Additionally, GDNF-RET signaling has been shown to induce estrogen receptor phosphorylation and transcriptional activity. In HER2-positive breast cancer, c-Src mediates a GDNF-dependent interaction between RET and HER2, generating multiple pro-cancer phenotypes in PDX models. In addition to the well-described estrogen regulation of RET expression, inflammatory cytokine signaling has been suggested as an alternate mechanism underlying RET overexpression

The inflammatory cytokine interleukin-6 (IL6) has been identified as a possible regulator of RET expression and signaling [75]. Using MCF-7 cell line models, it was demonstrated that GDNF treatment upregulates the mRNA expression of multiple cytokines, along with the release of IL6 protein. Importantly, it was noted that co-treatment with Fulvestrant showed an additive effect on IL6 release, suggesting that inflammation may act as part of the initial therapeutic response. Treatment of breast cancer cell lines with recombinant IL6 reproduced this effect, identifying IL6 as the first ER-independent regulatory system governing RET expression in breast cancer. Given the frequent combination of RET inhibitors with immunotherapeutic agents in the management of other tumor types (Additional file 1: Table S1), these findings may have critical implications for RET as a therapeutic target in ER-negative tumor subtypes (Fig. 3). However, the relationship between RET and inflammation in breast cancer remains an understudied area, urgently warranting further investigation.

## Targeting RET: past challenges and emerging therapeutic solutions

RET is a target for a plurality of multikinase inhibitors (MKIs) such as Cabozantinib, Sorafenib, Lenvatinib, and Vandetanib, most often used in the treatment of thyroid, liver, and renal cancers. While these have proven to be effective therapeutics in general, direct evidence of their antitumor effects via RET inhibition is lacking [90]. This, combined with challenges related to drug tolerance due to lack of specificity, has made the need for selective RET inhibitors for the treatment of RET-driven cancers a growing clinical interest. Accelerating genomic and functional insights, such as those reviewed above, provide a basis on which to examine the expanded use of highly selective RET inhibitors, including Pralsetinib (BLU-667) and Selpercatinib (LOXO-292) which are currently under preclinical and clinical investigation.

Multiple pre-clinical models of NSCLC and MTC have been used to highlight the possibility of potent RET inhibition by Pralsetinib, which was FDA-approved for the treatment of RET-altered thyroid cancers in December 2020. Pralsetinib exhibits a 10-to 100-fold reduction in IC_50_ against constitutively active RET fusion proteins compared to the MKIs Vandetanib and Cabozantinib in cell-free biochemical assays [2]. Importantly, Pralsetinib has in vitro inhibitory activity in cells expressing “gatekeeper mutations,” single amino acid substitutions which are known to confer MKI resistance. In addition, Pralsetinib exhibits in vivo functionality, both preventing and reversing tumor growth in models of PTC and RET fusion-driven NSCLC [91]. In the ARROW trial (focused on cancers of the lung and thyroid), Pralsetinib administration resulted in radiographic tumor size reduction in 90% of patients, including tumors harboring RET fusions. Increased efficacy was observed in patients previously treated with platinum-based chemotherapy. Additionally, anti-RET fusion activity is indiscriminate of fusion partner or CNS involvement, indicating that Pralsetinib may serve as an effective therapy for cancers driven by both RET alterations and by overexpression of the wild-type receptor [90]. While multiple ongoing clinical trials (Additional file 1: Table S1) are working to expand access to Pralsetinib in the treatment of colon and gastrointestinal cancers, to our knowledge, none are actively recruiting breast cancer patients at this time.

Selpercatinib (LOXO-292) is another small molecule RET inhibitor for which clinical trials are ongoing. In vitro, Selpercatinib has shown 60 to 1300-fold increased efficacy in targeting KIF5B-RET fusions when compared to Cabozantinib or Vandetanib [92]. In vivo, Selpercatinib treatment has produced significant reduction in tumor growth of CCDC6-RET fusion-positive NSCLC cells. Using an intracranial injection model for RET fusion-positive NSCLC brain metastasis in immunodeficient mice, it was reported that either Ponatinib (anti-BCR-ABL) or Selpercatinib treatment via intracranial administration significantly reduced tumor size. However, Selpercatinib showed antitumor activity at lower doses than those required for Ponatinib efficacy. Early clinical evidence shows a similarly positive patient response, with both MTC and NSCLC (with brain metastasis) patients showing enhanced radiographic response in comparison to Vandetanib or Cabozantinib treatment [92, 93]. Further, a recent case report highlights the efficacy of Selpercatinib when administered following an initial course of Tamoxifen therapy for the management of stage IV ER+, HER2− breast cancer [94]. Notably, the case reported by Watanabe et al. [94] exhibits a CCDC6-RET fusion in the absence of any other known breast cancer-associated mutations. While further study of the specific clinical role of RET fusions is required, the response of the patient described highlights an urgent need for the examination of RET alterations during planning of breast cancer therapeutics. Given the evidence suggesting a role for RET in breast cancer brain metastasis, the intracranial activity of modern RET inhibitors highlights importance of blood–brain barrier permeability, a design feature of multiple modern kinase inhibitors. Currently, 8 clinical trials of Selpercatinib are in various stages of recruitment and investigation. Most of these trials are focused on NSCLC, MTC, and PTC; however, one expanded access trial (NCT03906331; see Additional file 1: Table S1) is specifically interested in recruiting patients from other cancers, including breast cancer. Following the LIBRETTO-001 trial, Selpercatinib was approved by the US FDA for the treatment of RET-fusion positive NSCLC, RET-mutant driven MTC, and other RET-fusion driven thyroid cancers refractory to radioactive iodine therapy.

While the advent of highly selective RET inhibitors has been broadly successful, the progress made in this area of precision medicine has been met with distinct challenges. Subbiah et al. have reported cases of MTC (*n* = 1) and NSCLC (*n* = 1) in which therapeutic resistance developed after initially positive responses to Selpercatinib therapy [95]. Using cell-free DNA (cfDNA), novel non-gatekeeper mutations were detected in both patients, including G810C/S mutations at the solvent front and Y806C/N mutations in the RET kinase domain. Post-mortem examination of patients involved in clinical trials of Selpercatinib revealed multiple distinct subclones in Selpercatinib-resistant tumors, with mutations to G810 (G810S, G810C, and G810R) in common. This observation and subsequent case report has since been validated through preclinical modeling, in which patient-derived xenografts of CCDC6-RET-fusion positive NSCLC show multiple mutations at G810 in recurrent tumors [96]. While the RET inhibitors currently under examination have shown activity against anticipated gatekeeper mutations, the acquisition of novel RET mutations has emerged as a new challenge to drug design. Given the growing body of evidence suggesting a role for RET in endocrine resistance and estrogen-independent outgrowth, but uncertain function in primary breast cancers, further examination of gains in RET expression in luminal breast tumors during local recurrence or metastasis may prove to be a critical step in advancing of care for endocrine-refractory disease. Additionally, 10 of the 11 patients with a measurable central nervous system metastasis at enrollment in the LIBRETTO-001 trial showed a successful therapeutic response, highlighting the blood–brain barrier permeability of next generation kinase inhibitors and further supporting a role for RET inhibitor treatment in brain-metastatic breast cancer [97].

## Conclusions

In this review, we provide examples of the role for RET signaling in multiple facets of breast cancer progression, along with the known functions for RET overexpression and alterations across breast cancer subtypes. Some of these phenomena, such as the estrogen regulation of RET expression, are somewhat well-understood and have been studied extensively. In contrast, other topics presented here, including the potential for a relationship between RET signaling and inflammation, GDNF-mediated transcriptional activity, and novel RET fusion proteins, have yet to be characterized through repeated study. Additionally, the detection of RET overexpression in most of the models reviewed here is incidental, coinciding with the generation of Tamoxifen-conditioned, aromatase-overexpressing, or patient-derived cell lines. While few studies have utilized specific RET overexpression to date, we expect that the employment of such models, combined with the availability of highly specific and potent RET inhibitors, will enhance the precision of future in vitro and in vivo research related to RET in breast cancer.

The evidence presented here supporting the efficacy of RET-selective inhibitors, taken with the well-documented effects of RET signaling on multiple breast cancer phenotypes and clinical outcomes, highlights an emerging need to expand the study of RET as a specific target in breast cancer. While breast cancer is not a focus of the current clinical trials for RET-selective inhibitors, a wealth of preclinical data summarized here suggest that RET may have a critical role in the future of breast cancer therapy at varied stages of disease, in multiple drug combinations, and a variety of tumor subtypes.

## Supplementary Information

**Additional file 1: Table S1** Active clinical trials for RET-targeting agents in cancer.

## Data Availability

RNASeq data used to generate Fig. 2 are publicly available at https://leeoesterreich.org/resources.

## Abbreviations

RET: Rearranged during transfection
GDNF: Glial cell line-derived neurotrophic factor
GFL: GDNF family ligands
GFRα: GDNF family receptors
ERα: Estrogen receptor alpha
MAPK: Mitogen-activated protein kinase
PI3K: Phosphoinositide 3-kinase
AKT: Protein kinase B
mTOR: Mammalian target of rapamycin
PLC-γ: Phospholipase-C gamma
HER2: Human epidermal growth factor receptor 2
STAT: Signal transducer and activation of transcription
CCDC6: Coiled-coil domain-containing protein 6
NCOA4: Nuclear receptor coactivator 4
PDX: Patient-derived xenograft
FAK: Focal adhesion kinase
BCBM: Breast cancer brain metastasis
NSCLC: Non-small cell lung cancer
PTC: Papillary thyroid cancer
MTC: Medullary thyroid cancer
KIF5B: Kinesin-1 heavy chain
