# Supplementary material for: RET signaling in breast cancer therapeutic resistance and metastasis
Source: Breast Cancer Res. 2023 Mar 14;25:26. doi: 10.1186/s13058-023-01622-7 (PMC10015789; doi:10.1186/s13058-023-01622-7)
Supplement: Supplementary file 1 — Additional file 1: Table S1 Active clinical trials for RET-targeting agents in cancer. [file 13058_2023_1622_MOESM1_ESM.pdf]

Table 1. Active clinical trials for RET-targeting agents in cancer

Drug name (company)	ClinicalTrials.gov Identifier	MKI or RET-Selective	Disease Conditions	Treatments Combined	Phase Achieved	Associated Publications
**BOS172738 (Boston Pharmaceuticals)**	NCT03780517	RET-Selective	RET-altered MTC and NSCLC	NA	1	Schoffski et. al 2021
**Pralsetinib (Blueprint Medicine)**	NCT04302025	RET-Selective	RET-altered NSCLC	SOC chemotherapy + resection as required	2	
	NCT04760288		Medullary Thyroid Cancer	NA	3	NA
	NCT04697446		RET-Fusion positive NSCLC	NA	Retrospective	NA
	NCT04222972		RET-Fusion positive NSCLC	NA	3	NA
	NCT03037385		Multiple solid tumors	NA	2	NA
**Selpercatinib (Loxo Oncology/Eli Lilly)**	NCT04280081	RET-Selective	Medullary Thyroid Carcinoma, other tumors with RET alterations	NA	2	NA
	NCT04819100		NSCLC	NA	3	NA
	NCT04759911		RET-Altered Thyroid Cancer	SOC Surgery	2	NA
	NCT04320888		Multiple Cancers	NA	2	NA
	NCT04268550		RET-Fusion positive NSCLC	NA	2	NA
	NCT04211337		RET-Mutant Medullary Thyroid Carcinoma	NA	3	NA
	NCT04194944		RET-Fusion positive NSCLC	NA	3	NA
	NCT03906331		NSCLC, MTC, PTC, Breast, colon, pancreatic, and other cancers with RET alterations	NA	Not listed	NA
	NCT03899792		Pediatric CNS tumors	NA	2	NA
	NCT03157128		NSCLC, MTC, Colon cancer, other solid tumors	NA	2	NA
**TAS0953/HM06 (Helsinn)**	NCT04683250	RET-Selective	RET-Altered Solid Tumors	NA	2	NA

Table 1. Currently active or recruiting clinical trials of RET-targeting agents. RET is a target of numerous multikinase inhibitors, as displayed here. In contrast, RET-selective targeting agents are a newer class of RTK inhibitors, and are currently being examined for the treatment of a more specific set of cancers. SOC refers to standard-of-care therapy.

Table 1 (cont). Active clinical trials for RET-targeting agents in cancer

Drug name (company)	ClinicalTrials.gov Identifier	MKI or RET-Selective	Disease conditions	Treatments combined	Phase Achieved	Associated Publications
**Alectinib (Hoffmann- La Roche)**	NCT03194893	MKI (RET, ALK)	Multiple Cancers	Crizotinib	3	NA
**Anlotinib (Advenchen/Sino Biopharmaceutical)**	NCT04073550	MKI (VEGFR2/3, FGFR1-4, PDGFR, c-KIT, RET)	Small Cell Lung Cancer	NA	3	NA
	NCT04073537		Squamous NSCLC	Paclitaxel, Carboplatin	3	NA
**Cabozantinib (Exelixis)**	NCT04204850	MKI (c-MET, VEGFR2, AXL, RET)	Hepatocellular Carcinoma	NA	2	NA
	NCT04131543		NSCLC	NA	2	NA
	NCT03468985		NSCLC	Nivolumab	2	NA
	NCT03425201		Urothelial Cancer	Niraparib	2	NA
	NCT01639508		NSCLC	NA	2	Drilon et. al 2016
**Lenvatinib**	NCT04443309	MKI (VEGFR1, 2, 3; FGFR 1,2,3,4; PDGFR, c-KIT, RET)	Hepatocellular Carcinoma	Camrelizumab	2	NA
	NCT04425226		Hepatocellular Carcinoma	Pembrolizumab	NA	NA
	NCT04368078		Hepatocellular Carcinoma	Toripalimab	2	NA
	NCT04211168		Biliary Tract Cancers	Toripalimab	2	NA
	NCT04207086		Melanoma	Pembrolizumab	2	NA
	NCT03895970		Hepatobiliary Cancers	Pembrolizumab	2	NA
**Ponatinib (ARIAD pharmaceuticals)**	NCT02272998	MKI (BCR-ABL, VEGFR, PDGFR, FGFR, EPH, KIT, RET, TIE2, and FLT3)	Solid tumors with activating mutations of FGFR1, FGFR2, FGFR3, FGFR4, RET, or KIT	NA	2	NA
	NCT03838692		Medullary Thyroid Cancer	NA	2	NA
**Regorafenib (Bayer)**	NCT05048017	MKI (VEGFR1-3, TIE2, PDGFR, FGFR, KIT, RAF, RET)	Hepatocellular Carcinoma	Camrelizumab, Torpalimab, Pembrolizumab	2	NA
	NCT02736305		Ovarian Cancer		2	NA
**Sitravatinib (Mirati Therapeutics)**	NCT02219711	MKI (RET, CBL, Trk, DDR, TYRO3, AXL, MERTK)	Multiple solid tumors	NA	1	NA
**Sunitinib (Pfizer)**	NCT02691793	MKI (PDGFR, VEGFR, c-KIT, RET, CD114, CD135)	RET fusion-positive solid tumors	NA	4	NA
	NCT02450123		Refractory solid tumors (RET-altered or FGFR2-amplified)	NA	NA	NA
**TPX-0046 (TurningPoint Therapeutics)**	NCT04161391	MKI (RET, Src)	RET-Altered cancers (multiple)	NA	2	NA

Table 1. Currently active or recruiting clinical trials of RET-targeting agents. RET is a target of numerous multikinase inhibitors, as displayed here. In contrast, RET-selective targeting agents are a newer class of RTK inhibitors, and are currently being examined for the treatment of a more specific set of cancers. SOC refers to standard-of-care therapy.
